# A 16-year-old adolescent with a history of minor abdominal trauma diagnosed with a giant isolated primary splenic hydatid cyst: a case report

**DOI:** 10.1097/MS9.0000000000001851

**Published:** 2024-02-28

**Authors:** Abulfazl Vantankhah, Leila Ameri, Pegah Bahrami Taqanaki, Mohammad Jawed Bayat, Mahdi Parvizi Mashhadi

**Affiliations:** a Mashhad University of Medical Sciences; b Parsian Imaging Center; c Department of Pediatric Surgery, Mashhad University of Medical Sciences, Mashhad, Iran

**Keywords:** hydatid cyst, paediatric surgery, splenectomy

## Abstract

**Introduction:**

Hydatosis is a zoonotic parasitic disease caused by echinococcosis larval infection. South America, Africa, the Middle East, South Europe, India, and Australia are endemic to this disease. Splenic involvement is a rare and complicated hydatid disease presentation. A splenic hydatid cyst is an infrequent clinical occurrence, even in regions where the disease is endemic.

**Case presentation:**

A 16-year-old male, having a background of mild abdominal trauma and non-resolving dull abdominal pain attended a paediatric surgical outpatient office and following a thorough examination, was diagnosed with a giant solitary isolated splenic hydatid cyst. Subsequently, the patient received albendazole and underwent total splenectomy, necessitated by the considerable size of the cyst, classified as a giant.

**Clinical discussion:**

Splenic involvement of hydatid disease is a rare presentation (0.5–8%.). With the initial clinical finding often involving the accidental discovery of a palpable mass, the most frequently reported symptoms and signs include the presence of a palpable mass, fever, dull pain, or splenomegaly. Ultrasound and computed tomography are the most helpful tools for evaluating focal splenic diseases. The preferred treatment involves the use of antihelminthic drugs such as albendazole or mebendazole in conjunction with splenectomy. Total splenectomy is the preferred approach and is associated with decreased hospital stay, reduced healthcare costs, and a lower likelihood of recurrence.

**Conclusion:**

in endemic areas, in patients with splenic cysts, hydatidosis should be contemplated.

## Introduction

Highlights

- The study explores the necessity of splenectomy in the treatment of large and giant splenic hydatid cysts.
- This study sheds light on the abnormal manifestations of hydatid cysts and considers splenic hydatid cysts as a differential diagnosis in a patient with splenic enlargement and splenic cysts in an endemic area.
- The study thoroughly examines the manifestations associated with large and giant splenic hydatid cysts.

Hydatidosis is a persistent cyst-forming parasitic helminthic ailment affecting both humans and domestic as well as wild ungulates. It results from the infestation with the larval (metacestode) phases of dog tapeworms belonging to the genus Echinococcus within the family Taeniidae and is commonly denoted as echinococcosis^1^. The metacestode is a spherical, fluid-filled, unilocular cyst characterized by an inner germinal layer composed of cells, upheld by an acidophilic-staining, acellular, laminated membrane with variable thickness^2^. Infection occurs through the oral ingestion of eggs, typically resulting from contact with animal carriers, contaminated food, or water^3^. This ailment exhibits endemically in regions associated with cattle-rearing, encompassing South America, Africa, the Middle East, South Europe, India, and Australia. Iran stands out with the highest recorded incidence of the disease^4^. Hydatid cysts (HC) are the primary aetiology of splenic cysts globally, comprising 50–80% of all splenic cysts in endemic regions^5^.

A splenic hydatid cyst (SHC) is an infrequent clinical occurrence, even in regions where the disease is endemic^6^. This infrequency presents a diagnostic challenge for clinicians, especially in non-endemic regions. In areas where the disease is prevalent, it is imperative to incorporate the potential of SHC into the spectrum of differential diagnoses for individuals presenting with cystic lesions on the spleen until proven otherwise^4^. This case report has been reported in line with the 2023 criteria^7^.

## Case presentation

A 16-year-old adolescent attended a visit to an outpatient paediatric surgical office with a complaint of abdominal pain. The patient had encountered a minor blunt abdominal trauma while playing with peers 3 months ago. After the occurrence of the trauma, the patient visited an MD in a primary centre in the countryside of the city. Due to the physician’s diagnosis as a non-serious trauma, no further evaluation was performed and the patient was discharged afterward. In the following months, the patient experienced vague abdominal pain in the left hypochondrium. The patient stated that pain increased a little after the consumption of food. The patient had no history of fever, gastrointestinal reflux, dysphagia, and urinary symptoms such as haematuria. During the current visit, abdominal pain was characterized as non-sharp and predominantly localized in the left upper quadrant of the abdomen. The patient did not report any past medical history, including surgical and family history, and was not taking any medication.

**Figure 1:**
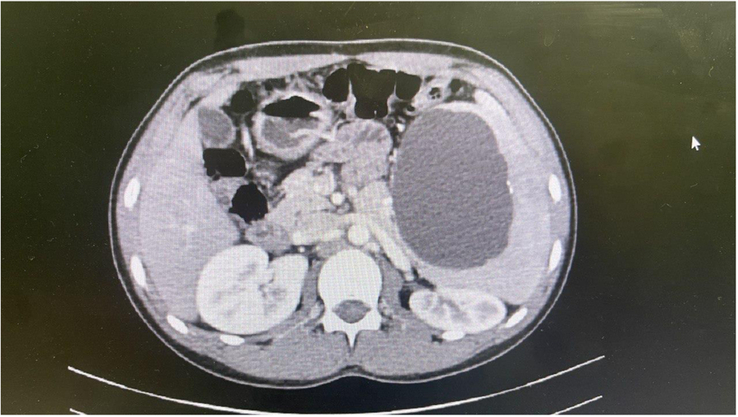
A computed tomography affirms the diagnosis of the giant splenic hydatid cyst. Several calcified foci are seen in the cyst wall. The germinal layer is intact and there is no sign of cyst rupture is visible. There are no signs of other organ involvement, most importantly liver and lung.

Upon physical examination, the patient, a male weighing 54 kg, with a BMI of 19.4, displayed no signs of a life-threatening condition. Abdominal examination revealed a tense mass under the costal margin, discernible both in superficial and deep examination. Given the mass’s location, indicative of potential spleen-related pathologies, an ultrasound examination was prescribed. The results revealed an enlarged spleen with a diameter of 193 mm, with a solitary, non-septate cystic compartment measuring 126×74 mm. Multiple hyperdense foci within the cyst were identified, suggesting calcification. Together, the ultrasound findings strongly suggest the presence of a hydatid cyst. A small amount of free intra-abdominal fluid was detected. The ultrasound examination revealed no involvement in other organs, including the liver. Subsequently, a computed tomography scan was conducted, affirming the diagnosis of the splenic hydatid cyst as the underlying cause (Fig. 1). The computed tomography (CT) scan did not show any signs of intrathoracic or intra-abdominal involvement of the hydatid cyst. Additional inquiry into the patient’s history revealed that the patient lived in rural countryside which was cramped with stray dogs. Blood samples were procured for laboratory analysis (Table 1), revealing mild leukocytosis in conjunction with eosinophilia.

**Table 1:** Laboratory data

Variables	Reference range	At the time of administration
Haemoglobin (g/dl)	12.0–16.0	13.3
Haematocrit (%)	36.0–46.0	40.6
White cell count (per μl)	3500–11 500	12 000
Platelet count (per μl)	150 000–400 000	452 000
Neutrophil (%)	50	40–80
Lymphocyte (%)	36	20–40
Eosinophil (%)	6	1–6

The patient was referred to a tertiary hospital. After consultation, a surgical intervention was scheduled for the patient. Given the stable condition and the absence of emergent circumstances, preoperative albendazole was initiated with a dose of 15 mg/kg for a duration of 2 weeks. The patient’s condition did not deteriorate during the following weeks.

**Figure 2:**
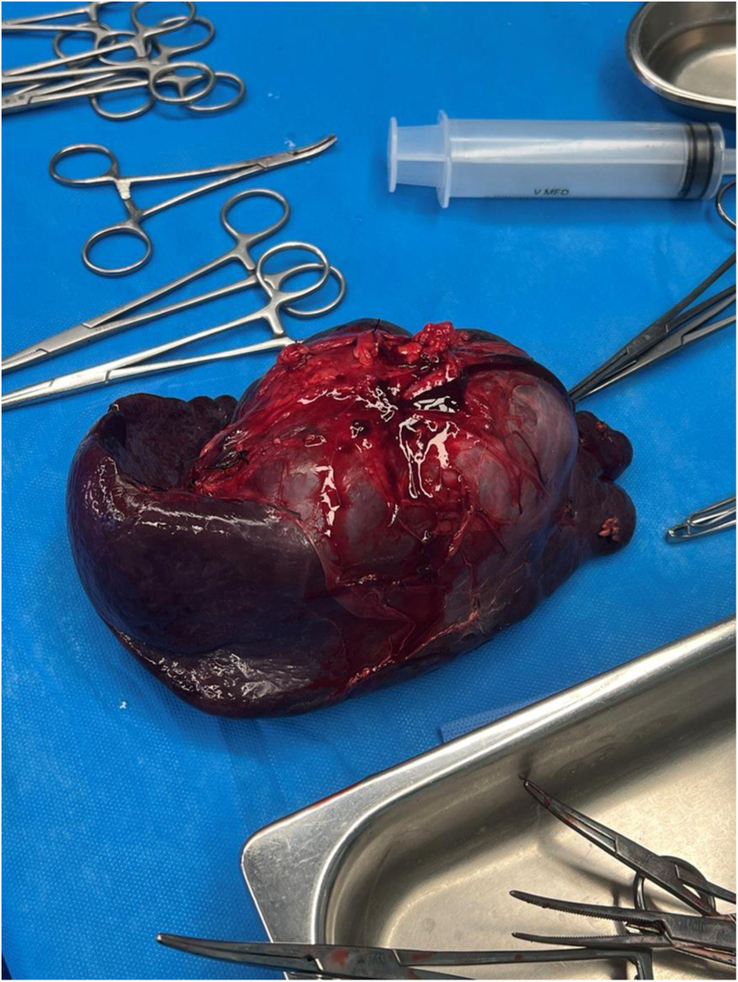
Resected Spleen following total splenectomy.

With the diagnosis of a giant splenic hydatid cyst, surgical intervention was undertaken. Following the induction of general anaesthesia, a transverse left subcostal incision was performed, and owing to numerous adhesions of the mass, the adhesions were released as much as possible. Next, splenocolic and splenorenal ligaments were ligated. Short gastric vessels were also released and ligated. After the splenic artery and vein were ligated separately, total splenectomy was undertaken, resulting in the removal of both the spleen and the intact cyst (Fig. 2). Intraoperative blood loss was minimal, and there was no necessity for blood transfusion, and the patient was in a favourable condition with stable vital signs. Pathological examination confirmed the diagnosis of a splenic hydatid cyst. Postoperatively, the patient did not experience any complications. Albendazole was prescribed for six months. A laboratory examination conducted 2 weeks after the initiation of albendazole revealed only a slight increase in aminotransferase levels, well below the discontinuation threshold. Subsequent laboratory follow-ups were scheduled every 2 weeks for 3 months, followed by monthly assessments for an additional 3 months. A post-surgery ultrasonography performed three months after the procedure indicated no signs of recurrence.

## Discussion

HCs involving the spleen are extremely rare, with documented frequency ranging from 0.5 to 8%. SHC may manifest as isolated or a component of disseminated disease resulting from a ruptured hepatic HC^8^. The suggested routes for primary SHC include arterial paths passing through the liver and lung or migration through the portal circulation. Secondary SHC typically occurs following systemic or intraperitoneal spread subsequent to a hepatic HC rupture. The largest reported cyst, documented in Australia and containing 57 l of fluid^4^, showcases the rapid growth of HCs in unusual sites like the spleen^9^. Isolated splenic involvement is rare^10^, with ~20–30% of cases showing simultaneous involvement of the liver and/or lungs^4^.

Remarkably, up to 30% of cases of SHC are coincidental findings in asymptomatic patients, with the initial clinical finding often involving the accidental discovery of a palpable mass, predominantly in the left hypochondrium or occasionally in the epigastrium. The most frequently reported symptoms and signs include the presence of a palpable mass, fever, dull pain, or splenomegaly. In some cases, patients may experience complications such as anaphylactic shock due to cyst rupture, secondary infections, or the formation of a fistula with adjacent hollow organs. Additional symptoms might include dyspepsia, constipation due to pressure on the colon, and dyspnoea resulting from the elevation of the left diaphragm. Fistula formation with the colon or perforation into the diaphragm or bronchial tree is also possible^4^.

The primary challenge in diagnosing SHC lies in distinguishing it from other cystic lesions in the spleen. SHCs, typically solitary, share imaging characteristics similar to hepatic HCs. Among the diagnostic imaging techniques, ultrasound (US) and CT are the most helpful tools for evaluating focal splenic diseases. The US is particularly beneficial in the early stages, of cystic lesions, detecting daughter cysts, hydatid membranes, and hydatid sand. CT, with sensitivity rates ranging from 95 to 100% compared to the US, excels in assessing the quantity, size, and location of hepatic and extrahepatic cysts, and could prove advantageous in monitoring lesions during treatment progress and identifying recurrences. The differential diagnosis for such lesions encompasses epidermoid cysts, pseudocysts, large solitary abscesses or haematomas, intra-splenic pancreatic pseudocysts, lymphangiomas, hemangiomas, and cystic spleen neoplasms^4,11,12^.

In the management of SHCs, the preferred treatment involves the use of antihelminthic drugs such as Albendazole or Mebendazole in conjunction with splenectomy. Splenectomy stands as the conventional and optimal treatment choice due to its associated low morbidity and mortality rates. Total splenectomy is recommended in cases where the spleen is nearly entirely occupied by the HC, when dealing with giant cysts (HCs with a diameter of 10 cm or larger are referred to as “giant” cysts^13^) at an elevated risk of rupture or compression of vital structures, or when multiple cysts are present, particularly in more central or hilar locations. While total splenectomy may entail a higher risk of morbidity and mortality, it is nonetheless recommended in poor and underdeveloped countries. Alternative conservative modalities include cyst enucleation, partial cystectomy, percutaneous aspiration irrigation and re-aspiration (PAIR), and the intraoperative application of scolicidal agents^4,8,14–17^. Total splenectomy is the preferred approach due to its association with specific peri-operative therapeutic measures. This method ensures a decreased hospital stay, reduced healthcare costs, and a lower likelihood of recurrence. Patients undergoing spleen-preserving surgery tend to experience a longer average hospital stay, primarily attributable to postoperative complications such as abscess formation in the residual cavity and postoperative haemorrhage when compared to those who undergo total splenectomy^18^. Furthermore, there is no statistically significant difference in recurrence rates observed between splenectomy and spleen-sparing surgery^19^. Laparoscopic splenectomy is deemed suitable for SHD exceeding 5 cm in size, especially those surpassing 10 cm. This approach is particularly indicated for superficial cysts at risk of rupture or located in the central segment of the spleen, cysts compressing vital structures, those presenting with secondary infection or haemorrhage, and instances involving multiple cysts resulting from either direct inoculation or secondary spread from other organs^15^.

Albendazole exhibits significantly greater efficacy compared to mebendazole in the treatment of HCs. It is preferred to administer albendazole at an average dosage of 10–15 mg/kg/day, divided into two doses, accompanied by a fat-rich meal to enhance its bioavailability. The presently recommended treatment duration is 3–6 months, with a 14-day break. Pre-surgery administration of albendazole may facilitate the complete removal of the germinal layer. Ideally, in accordance with WHO guidelines, preoperative prophylaxis with albendazole should commence one month prior to surgery, or at the very least, 4 days before the surgical procedure. This approach aims to stabilize cysts, reduce tension within the cyst, and minimize the risks of anaphylaxis and recurrence^4,20,21^. While albendazole is generally well-tolerated by the majority of patients, it is associated with certain adverse effects, such as nausea, vomiting, abdominal pain, diarrhoea, dizziness, headache, and gastrointestinal disturbances. Additionally, albendazole use may result in abnormalities in liver functions, leukopenia, and haematuria^22^.

Cystic echinococcosis has the potential to relapse several years post-treatment. A follow-up period of at least 5 years is necessary to assess and ascertain the occurrence of local recurrences^23^. In the case of splenic hydatidosis, Recurrence rates appear to be notably low after splenectomy, but there is a potential for higher rates with spleen-sparing operations or when additional cysts are removed from other organs^4^.

### Limitations

The lack of serological examinations is one of the major pitfalls in the current report. Furthermore, there is a deficiency in data concerning the patient’s prior visits to the initial physician. The poor adherence of the patient has led to a shortfall in the completion of the scheduled follow-up laboratory and imaging examinations.

## Conclusion

In endemic areas, physicians should consider Hydatidosis in patients with splenic cysts, particularly in cases with a history of living in rural areas and exposure to dogs and other livestock materials, as adding this ailment to their differential diagnosis checklist can aid physicians in approaching patients with this rare and challenging encounter.

## Ethical approval

Not applicable.

## Consent

Written informed consent was obtained from the patient for publication of this case report and accompanying images. A copy of the written consent is available for review by the Editor-in-Chief of this journal on request.

## Source of funding

This research did not receive any specific grant from funding agencies in the public, commercial, or not-for-profit sectors.

## Author contribution

Study concept or design: M.P.M., A.V. Data collection: A.V., L.A., P.B.T., M.J.B.T. Supervision: M.P.M. Writing the paper: A.V., L.A., P.B.T., M.J.B.T. Revision: M.P.M., A.V.

## Conflicts of interest disclosure

Not applicable.

## Research registration unique identifying number (UIN)

9773.

## Guarantor

Mahdi Parvizi Mashhadi.

## Data availability statement

Data sharing is not applicable to this article.

## Provenance and peer review

Not commissioned, externally peer-reviewed.
